# N-Acetyl Cysteine Depletes Reactive Oxygen Species and Prevents Dental Monomer-Induced Intrinsic Mitochondrial Apoptosis *In Vitro* in Human Dental Pulp Cells

**DOI:** 10.1371/journal.pone.0147858

**Published:** 2016-01-25

**Authors:** Yang Jiao, Sai Ma, Yirong Wang, Jing Li, Lequn Shan, Qian Liu, Ying Liu, Qian Song, Fan Yu, Haohan Yu, Huan Liu, Li Huang, Jihua Chen

**Affiliations:** 1 State Key Laboratory of Military Stomatology, Department of Prosthodontics, School of Stomatology, the Fourth Military Medical University, Xi’an, PR China; 2 Shaanxi Key Laboratory of Military Stomatology, Xi’an, Shaanxi, PR China; 3 State Key Laboratory of Military Stomatology, Department of Operative Dentistry and Endodontics, School of Stomatology, the Fourth Military Medical University, Xi’an, PR China; 4 Department of Orthopaedic Oncology, Xijing Hospital Affiliated to the Fourth Military Medical University, Xi’an, PR China; 5 Department of Orthopaedic Surgery, Tangdu Hospital, the Fourth Military Medical University, Xi’an, PR China; 6 State Key Laboratory of Military Stomatology, Department of Orthodontics, School of Stomatology, the Fourth Military Medical University, Xi’an, PR China; 7 State Key Laboratory of Military Stomatology, Department of General and Emergency, School of Stomatology, the Fourth Military Medical University, Xi’an, PR China; National Cheng Kung University, TAIWAN

## Abstract

**Purpose:**

To investigate the involvement of intrinsic mitochondrial apoptosis in dental monomer-induced cytotoxicity and the influences of N-acetyl cysteine (NAC) on this process.

**Methods:**

Human dental pulp cells (hDPCs) were exposed to several dental monomers in the absence or presence of NAC, and cell viability, intracellular redox balance, morphology and function of mitochondria and key indicators of intrinsic mitochondrial apoptosis were evaluated using various commercial kits.

**Results:**

Dental monomers exerted dose-dependent cytotoxic effects on hDPCs. Concomitant to the over-production of reactive oxygen species (ROS) and depletion of glutathione (GSH), differential changes in activities of superoxide dismutase, glutathione peroxidase, and catalase were detected. Apoptosis, as indicated by positive Annexin V/propidium iodide (PI) staining and activation of caspase-3, was observed after dental monomer treatment. Dental monomers impaired the morphology and function of mitochondria, and induced intrinsic mitochondrial apoptosis in hDPCs via up-regulation of p53, Bax and cleaved caspase-3, and down-regulation of Bcl-2. NAC restored cell viability, relieved oxidative stress and blocked the apoptotic effects of dental monomers.

**Conclusions:**

Dental monomers induced oxidative stress and mitochondrial intrinsic apoptosis in hDPCs. NAC could reduce the oxidative stress and thus protect hDPCs against dental monomer-induced apoptosis.

## Introduction

Owing to their physical and aesthetic properties, resin-based materials are routinely used to restore the structure and function of teeth [1, 2]. However, residual monomers released from resin restorations as a result of incomplete polymerization could have irritating effects on the oral tissues. Several dental monomers, including 2-hydroxyethyl methacrylate (HEMA), methyl methacrylate (MMA), and triethylenglycol dimethacrylate (TEGDMA), have been identified as cytotoxic molecules that disrupt the stable redox balance and result in oxidative stress [3, 4]. The imbalanced redox state of the cells, characterized by the over-production of reactive oxygen species (ROS) and depletion of glutathione (GSH), has been shown to induce cell death via apoptosis [4–6]. However, the exact and detailed mechanism underlying dental monomer-induced apoptosis is still largely unknown. Apoptosis can be triggered by various signals. In particular, ROS can induce oxidative DNA damage, which can subsequently upregulate p53, and thus trigger intrinsic mitochondrial apoptosis by shifting the balance in the Bcl-2 family [7–9]. Thus, one of the purposes of the present study is to investigate the possible involvement of mitochondrial intrinsic apoptotic pathway in dental monomer-induced cytotoxicity.

Novel strategies are needed to reduce the adverse effects of dental materials. To design such strategies, it is necessary to understand the exact mechanisms by which these materials induce cell death and to find strategies to decrease or eliminate their toxicities. N-acetyl cysteine (NAC) has played a well-documented role in detoxifying dental monomers and resinous materials [10–12]. However, the influences of NAC on dental monomer-induced apoptosis have not been elucidated yet. Thus, the second and the most important purpose of the present study is to investigate the influences of NAC on dental monomer-induced apoptosis.

## Materials and Methods

### Cell cultures and cell proliferation assay

Human dental pulp cells (hDPCs) were derived from primary culture, as described in our previous study [12]. Briefly, hDPCs were isolated from the dental pulp tissues of non-carious third molars extracted from young healthy patients (18–25 years old), according to a protocol that was verbally approved by the Ethics Committee of the Fourth Military Medical University (approval number: 15–20) with written informed consent obtained from all subjects. Extracted teeth were delivered to the cell culture laboratory in isolation medium containing alpha-modified Eagle’s medium (α-MEM; Gibco BRL Division of Invitrogen, Gaithersburg, MD, USA) supplemented with 10% fetal bovine serum (Gibco), 100 units/mL penicillin, and 100 mg/mL streptomycin. Upon arrival to the laboratory, the dental pulp was minced and digested in a solution containing 3 mg/mL type I collagenase and 4 mg/mL dispase (Gibco) at 37°C for 2 h [13]. Single-cell suspensions were obtained by passing the cells through a 70-mm strainer (BD Falcon, Franklin Lakes, NJ, USA) and cultured in α-MEM supplemented with 10% fetal bovine serum, 100 units/mL penicillin, and 100 mg/mL streptomycin. Media were changed every 3 days in 5% CO_2_ at 37°C.

Firstly, Cell Counting Kit-8 (CCK-8) (Beyotime Biotechnology, China) was used to determine the influences of dental monomers on the viability of hDPCs. Briefly, hDPCs at the second passage were seeded into 96-well culture plates at 5 × 10^3^ cells/well and incubated at 37°C and 5% CO_2_ for approximately 24 h. When the cells reached 80% confluence, media were removed. Cells were treated with media containing 2-hydroxyethyl methacrylate (HEMA; 1, 2.5, 5, 7.5, or 10 mM), methyl methacrylate (MMA; 1, 2.5, 5, 7.5, or 10 mM), or triethylenglycol dimethacrylate (TEGDMA; 1, 2.5, 5, 7.5, or 10 mM) for 24 h. All the tested dental monomers were purchased from Sigma-Aldrich (St. Louis, MO, USA) and dissolved in complete media. CCK-8 solution was added, and the cells were incubated at 37°C for another 4 h. Absorbance of the colored solution was measured with a microplate reader (Bio-Rad Laboratories, Hercules, CA) at a wavelength of 450 nm. Cells in the control group were treated with culture medium without dental monomers. Here, we found the lowest concentration for each monomer that could induce a significant decrease in cell viability (HEMA: 1mM; MMA: 5mM; TEGDMA: 1mM). In the following study evaluating dental monomer-induced oxidative stress and apoptosis, 1 mM HEMA, 5 mM MMA and 1 mM TEGDMA were used.

Human DPCs were also exposed to 1mM HEMA, 5mM MMA and 1mM TEGDMA in the absence or presence of 10 mM NAC for 24, 48, 72 or 96 h to test the influence of NAC on monomer-induced cytotoxicity. NAC (Sigma; St Louis, MO, USA) was dissolved in complete media to a final concentration of 10 mM as previously described [14]. After co-treatment of NAC and monomer for 24 h, the morphology of the cultured cells was observed and documented by phase contrast microscopy (1X70, Olympus, Tokyo, Japan). Results for CCK-8 assay were plotted as the mean ± SD of three independent experiments with six determinations per sample for each experiment.

### Measurement of intracellular reactive oxygen species (ROS)

Levels of intracellular ROS were measured with an ROS assay kit (Beyotime Biotechnology, China). After treatment with dental monomers (1mM HEMA, 5mM MMA and 1mM TEGDMA) in the absence or presence of 10 mM NAC for 6 h, hDPCs were treated with dihydrodichlorofluorescein diacetate (DCF-DA), incubated in serum-free media at 37°C for 20 min, harvested in phosphate-buffered saline (PBS), and analyzed in a FACSCalibur flow cytometer (BD Biosciences). Results were plotted as the mean ± SD of three determinations per sample for each experiment, with 10,000 cells being analyzed for each sample.

### Biochemical assays

Human DPCs were seeded into a 6-well plate at a density of 1 × 10^5^ cells/well, incubated for 24 h, and treated with dental monomers (1mM HEMA, 5mM MMA and 1mM TEGDMA) in the absence or presence of 10 mM NAC. After 24 h, the cells were collected and resuspended in the buffer for lyticase (Sigma, USA) for 30 min at 30°C. Then the lysate was centrifugated (10,000×g, 5 min, 4°C), and the supernatant was collected. Total protein concentrations of parallel samples were measured using a BCA Protein Assay Kit (Beyotime Biotechnology, China). The supernatant was aliquoted and stored at -80°C. All experiments were repeated three times.

Contents of total glutathione (total GSH), reduced glutathione (GSH) and oxidized disulfide (GSSG) were measured using a commercially available kit (Beyotime Biotechnology, China) according to the manufacturer’s protocol.

After the supernatant was collected, total GSH was assayed using the 5,5-dithio-bis(2-nitrobenzoic) acid (DTNB)-GSSG reductase recycling. GSSG was measured by measuring 5-thio-2-nitrobenzoic acid (TNB) which was produced from the reaction of reduced GSH with DTNB. Then the fluorescence of the samples was evaluated using a microplate reader (Bio-Rad Laboratories, Hercules, CA) at 405 nm. Total GSH and GSSG concentration were normalized to protein contents. The concentration of reduced GSH in the sample was obtained by subtracting GSSG from total GSH. The experiment was done in triplicate and repeated for three times.

Lipid peroxidation was assayed by the measurement of malondialdehyde (MDA), according to the instructions of lipid peroxidation MDA assay kit (Beyotime Biotechnology, China). One hundred μL supernatant was mixed with 100 μL MDA working solution and boiled at 100°C for 15 min. Samples were cooled down to room temperature in a water bath and then centrifuged at 1,000g for 10 min. The absorbance at 532 nm was measured in a 96-well plate (200 μL/well) with a microplate reader (Bio-Rad Laboratories, Hercules, CA). The results of MDA assay were expressed as micromoles of MDA per milligram of protein (nmol/mg protein). The experiment was done in triplicate and repeated for three times.

The superoxide dismutase (SOD) activity was quantified with the SOD Assay Kit (Beyotime Biotechnology, China). The 50% inhibitory activity of SOD was determined by the colorimetric method and quantified by measuring the absorbance at 450 nm using a microplate reader (Bio-Rad Laboratories, Hercules, CA). The activity of glutathione peroxidase (GPx) was quantified in the supernatant by using a GPx Assay Kit (Beyotime Biotechnology, China) and measuring the degree of reducing absorption of NADPH at 340 nm. The catalase (CAT) activity was quantified by using the CAT Assay Kit (Beyotime Biotechnology, China). The activities of SOD, GPx and CAT were normalized to protein contents. Results were plotted as the mean ± SD of three independent experiments with three determinations per sample for each experiment.

### Quantification of apoptotic cells by flow cytometry

Dental monomer-induced cell apoptosis and the protective effects of NAC were quantified with an Annexin V-FITC apoptosis detection kit (Beyotime Biotechnology, China). Human DPCs were seeded into a 6-well plate at 1×10^5^ cells/well, incubated for 24 h, and treated with HEMA (0-1-5-10 mM), MMA (0-5-10-15 mM) or TEGDMA (0-1-5-10 mM), in the absence or presence of 10 mM NAC. Untreated cells served as the negative control. After culturing for 24 h, hDPCs were collected and washed with PBS, gently resuspended in Annexin V binding buffer, and incubated with Annexin V-FITC/propidium iodide (PI). Flow cytometry was performed using flow cytometric analysis (Becton-Dickinson FACScan). Results were plotted as the mean ± SD of three determinations per sample for each experiment, and 10,000 cells were analyzed for each sample.

### Analysis of caspase-3 activity

To investigate caspase-3 activation after treatment with dental monomers in the absence or presence of NAC, the Caspase-3 Colorimetric Assay Kit (Beyotime Biotechnology, China) was used. Briefly, 1×10^6^ cells treated with dental monomers (1mM HEMA, 5mM MMA and 1mM TEGDMA) with or without 10 mM NAC for 24 h were collected and lysed in a lysis buffer. Cell lysates were tested for protease activity by using Asp-Glu-Val-Asp-pNA (DEVD-pNA), a tetrapeptide p-nitroanilide substrate. Triple wells were used for each group. After incubation with this substrate for 2 h, the absorbance was measured at 405 nm with a microplate reader (Bio-Rad Laboratories, Hercules, CA). Caspase enzymatic activities in cell lysates were normalized to protein contents measured in a BCA Protein Assay Kit. The results were presented as means ± SD of three independent experiments.

### Intercellular ATP level determination

ATP level was measured by using a firefly luciferase based ATP Assay Kit (Beyotime Biotechnology, China) according to the manufacturer’s instructions. Cells were seeded into a 6-well plate at a density of 1×10^5^ cells/well. Then the cells were treated with dental monomers (1mM HEMA, 5mM MMA and 1mM TEGDMA) in the absence or presence of 10 mM NAC for 24 h. After rinsed with PBS, the cells were schizolysised by solution and then centrifuged at 12,000g at 4°C for 15 min and the supernatant was collected. After 100 μL of the supernatant was mixed with 100 μL of ATP detection solution, intensity readings were taken with a microplate reader (Bio-Rad Laboratories, Hercules, CA). The ATP concentrations in samples were calculated using an ATP standard curve and the protein concentration of each treatment group was determined using the BCA protein assay kit. The cellular ATP levels were expressed as nmol/mg protein.

### Assessment of mitochondrial membrane potential (MMP)

The fluorescent dye JC-1 (Beyotime Biotechnology, China) was used to detect cells with collapsed MMP. Briefly, cells cultured in α-MEM were exposed to dental monomers (1mM HEMA, 5mM MMA and 1mM TEGDMA) in the absence or presence of 10 mM NAC for 24 h and incubated with the JC-1 staining solution (5 mg/ml) for 20 min at 37°C. Cells were rinsed twice with JC-1 staining buffer. The fluorescence intensity (FI) of JC-1 aggregates was detected at an excitation/emission wavelength ratio of 525 nm/590 nm, and the FI of the JC-1 monomers was measured at an excitation/emission wavelength ratio of 490 nm/530 nm by flow cytometry (Becton-Dickinson FACScan). All results were plotted as the mean ± SD of three determinations per sample for each experiment, and 10,000 cells were analyzed for each sample.

### Western blot analysis

Human DPCs cultured in α-MEM were exposed to dental monomers (1mM HEMA, 5mM MMA and 1mM TEGDMA) in the absence or presence of 10 mM NAC for 24 h (*n* = 3) and lysed in RIPA buffer. Protein contents of the lysed cells were measured with the BCA Protein Assay Kit. Extracted proteins were loaded on 10% sodium dodecyl sulfate polyacrylamide gels, transferred to polyvinylidene fluoride membranes (Bio-Rad), and blocked with 5% nonfat milk powder. Membranes were incubated overnight with the following primary rabbit anti-human antibodies (Cell Signaling Technology, Danvers, MA): anti-Bcl-2, anti-Bax, anti-p53, anti-cleaved caspase-3, and actin. Membranes were incubated with goat anti-rabbit secondary antibodies. Protein signals were visualized by using the ECL Western Blotting Detection System (GE Healthcare, Piscataway, NJ, USA). Protein expression levels were normalized to actin by using Image-Pro Plus 5.0 Software (Media Cybernetics Inc., Bethesda, MD, USA).

### Analysis of cytosolic cytochrome c (Cyto C) release

After hDPCs were exposed to dental monomers (1mM HEMA, 5mM MMA and 1mM TEGDMA) without or with 10 mM NAC for 24 h, the release of CytoC from mitochondria to the cytoplasm was investigated. Cells were resuspended in 1.5 mL of cold Mito-Cyto Buffer (Applygen Biotechnology, China), lysed, and centrifuged. The supernatant was centrifuged again at 12,000 g for 10 min to separate mitochondria (in pellet) from the cytoplasm (in supernatant). Cytosolic Cyto C in the supernatant was quantified with an ELISA kit (Westang Biotechnology, China), using Cyto C (0–2000 pg/mL) as a standard. Data are expressed as the total amount of intracellular solubilized Cyto C. Cyto C levels were normalized to protein content measured by a BCA Protein Assay Kit. All results were plotted as the mean ± SD of three determinations per sample for each experiment.

### Immunofluorescence (IF)

Human DPCs were seeded into a 6-well plate at a density of 1×10^5^ cells/well and exposed to dental monomers (1mM HEMA, 5mM MMA and 1mM TEGDMA) without or with 10 mM NAC for 24 h. The cells were then fixed with a modified Zamboni's fixative (4% paraformaldehyde and 0.19% picric acid in PBS, pH 7.4) for 30 min at room temperature as described before [15]. Cells were washed twice with PBS, preincubated in the same buffer containing 0.3% Triton X-100 (Amresco, USA), and blocked with 0.1% BSA (Amresco, USA) in PBS for 1h. The cells were incubated with primary antibodies (rabbit anti-Bax, 1:200, Beyotime Biotechnology, China; and mouse anti-Cytochrome C, 1:50, Boster Biotechnology, China) overnight at 4°C. After three washes with PBS, cells were incubated with secondary antibodies (Alexa Fluor 594 donkey-rabbit, 1:400; and Alexa Fluor 488 donkey-mouse, 1:200; Life Technologies, Carlsbad, CA) for 30 min at 37°C. Cells were then washed in PBS and further incubated with 10 mg/ml DAPI (Sigma) at room temperature for 10 min. Fluorescent images were obtained by laser scanning confocal microscopy (Keyence Co., Osaka, Japan).

### Transmission Electron Microscopy (TEM)

Human DPCs were initially collected after exposure to dental monomers (1mM HEMA, 5mM MMA and 1mM TEGDMA) without or with 10 mM NAC for 24 h, and fixed with 2.5% glutaraldehyde in 0.1 M cacodylate buffer for 2 h and post fixed with a solution of 1% osmium tetroxide in 0.1 M cacodylate buffer. The cells were then embedded in epoxy resins after a graded-ethanol serial dehydration step. The embedded cells were sectioned into ultrathin slices, stained by uranyl acetate solution and lead citrate, and then observed with a transmission electron microscope Tecnai G2 Spirit BioTWIN electron microscope (FEI Company, Eindhoven, The Netherlands).

### Statistical analysis

The SPSS 18.0 software package (SPSS Inc., Chicago, IL, USA) was used to perform statistical analyses. Data was analyzed by one-way analysis of variance (ANOVA), followed by the Tukey’s test. The statistical significance level was set at *P* = 0.05 for all tests.

## Results

### Influences of dental monomers and NAC on cell viability and cell morphology

**Fig 1:**
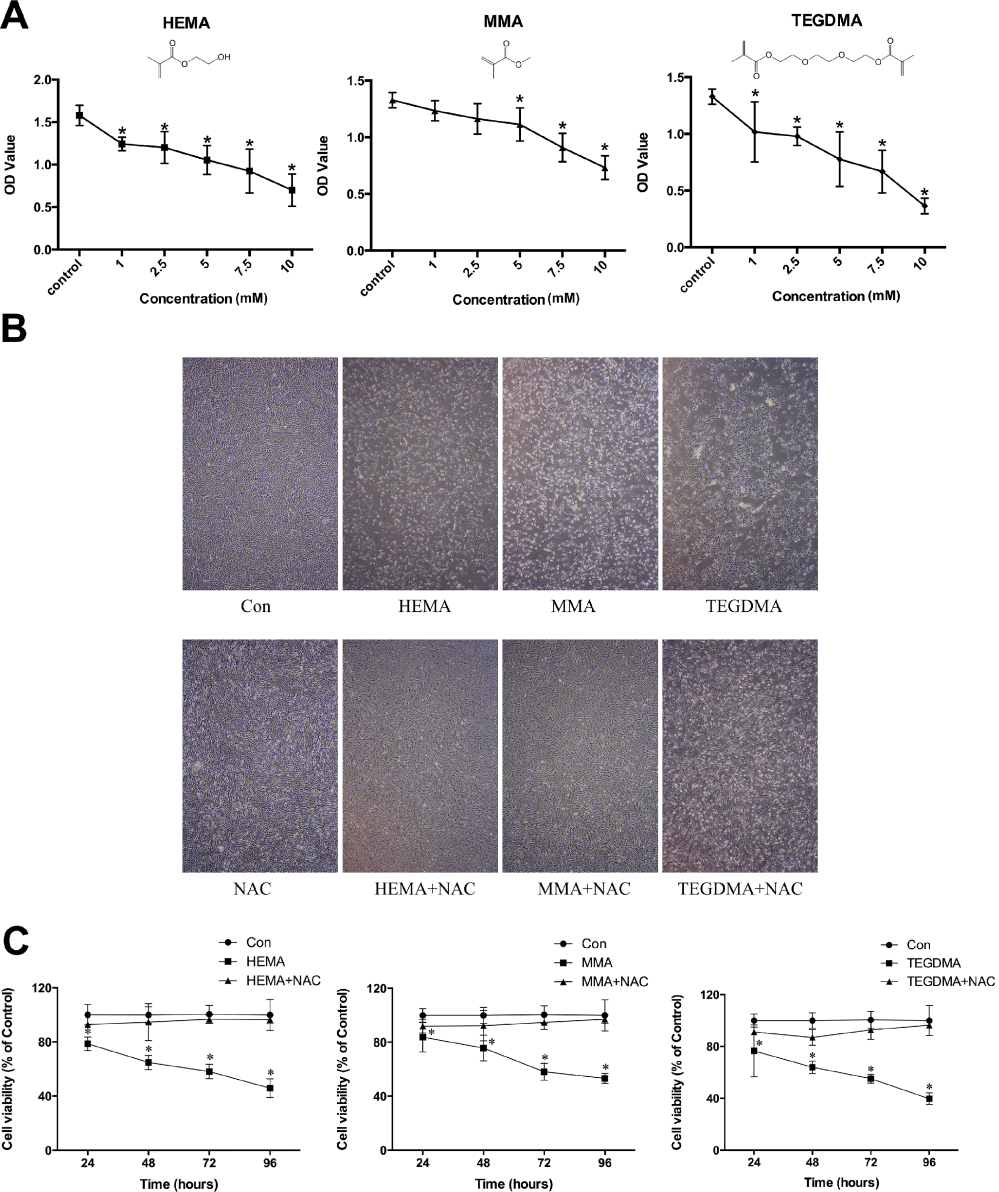
Dental monomers induced cytotoxic effects in hDPCs. A. Cell viability of hDPCs after dental monomer treatment (HEMA: 1–2.5-5-7.5–10 mM; MMA; 1–2.5–5–7.5–10 mM; or TEGDMA: 1–2.5-5-7.5–10 mM) for 24 h, as analyzed by the CCK-8 assay. B. Morphological changes in hDPCs after treatment with dental monomers (1mM HEMA, 5mM MMA or 1mM TEGDMA) in the absence or presence of 10 mM NAC. C. Cell viability of hDPCs after exposure to dental monomers (1mM HEMA, 5mM MMA or 1mM TEGDMA) without or with 10 mM NAC. Data represent the mean ± SD of three independent experiments (*n* = 6). *P < 0.05 vs. control group by one-way ANOVA.

The CCK-8 assay results revealed that treatment of hDPCs with dental monomers for 24 h decreased cell viability in a dose-dependent manner (Fig 1A). Compared to the control group, groups that were treated with HEMA (≥1 mM), MMA (≥5 mM), or TEGDMA (≥1 mM) exhibited significantly lower cell viability. In subsequent experiments to investigate influences of dental monomers on intracellular redox balance and apoptosis, hDPCs were treated with dental monomers at the lowest concentrations that can induce significant difference on cell viability. Fig 1B showed representative cell micrographs after treatment with various dental monomers in the absence or presence of NAC. Cells in the control group exhibited normal growth and typical spindle appearance. However, many cells became round after treatment with dental monomers, and cell population density was further reduced. Co-treatment of NAC with monomers restored cell density and preserved the normal morphology of cells. Furthermore, the long-term effects of dental monomers and NAC on cell viability were also tested. As shown in Fig 1C, a time-dependent decrease in cell viability was observed in cells exposed to 1 mM HEMA after 24 h, 48 h, 72 h and 96 h (all *P* < 0.05 as compared to control group). Likewise, similar time-dependent inhibitory effects were observed in cells exposed to 5 mM MMA and 1 mM TEDGMA (all *P* < 0.05 as compared to control group). Quite the reverse, the presence of NAC could significantly restore the cell viability (all *P* > 0.05 as compared to control group) for all the studied time periods.

### Influences of dental monomers on intracellular redox balance and the protective effects of NAC on hDPCs

**Fig 2:**
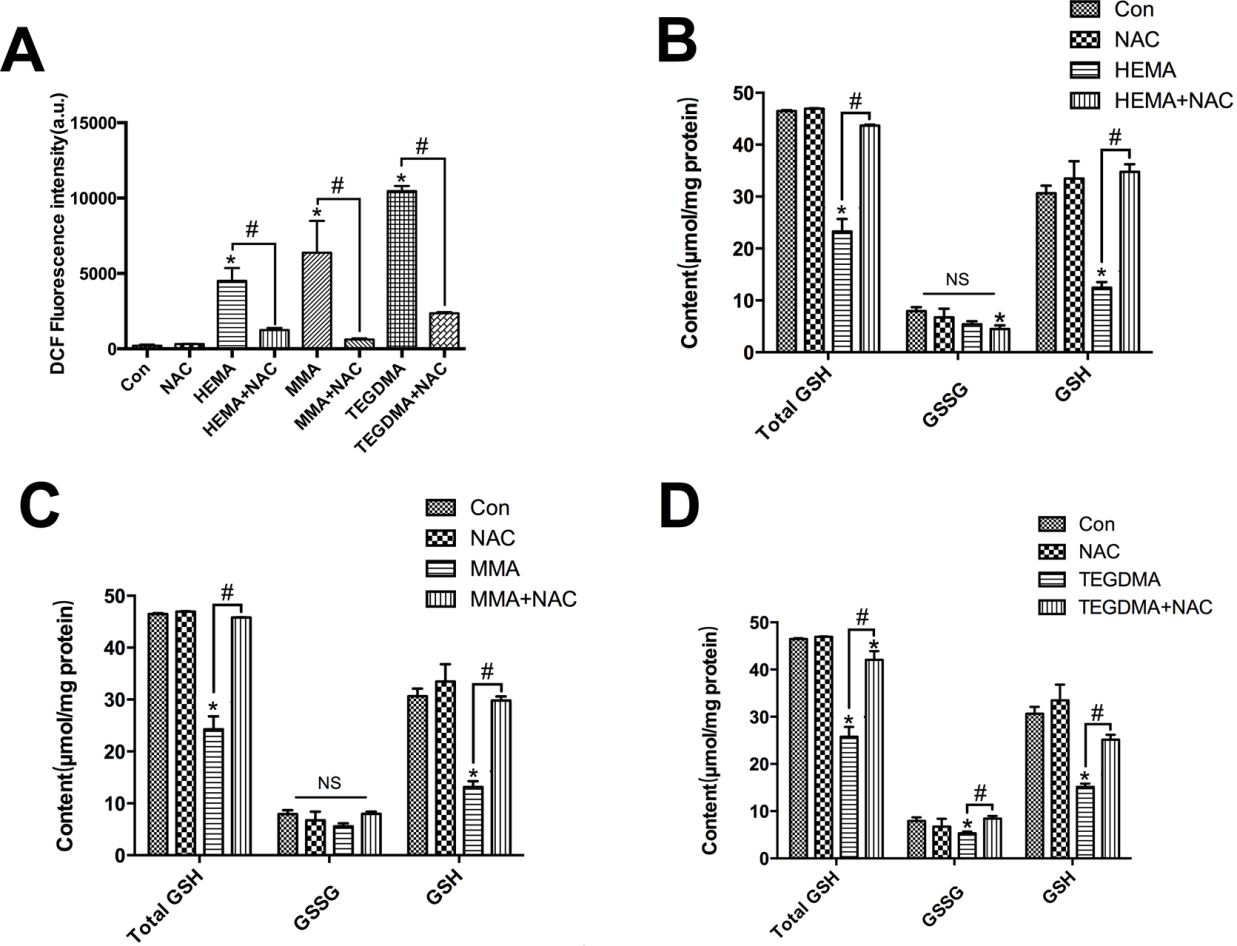
Dental monomers induced over-production of ROS and depletion of GSH. A. Intracellular ROS levels in hDPCs exposed to dental monomers (1mM HEMA, 5mM MMA or 1mM TEGDMA) in the absence or presence of 10 mM NAC for 6 h. B–D. Contents of GSH, GSSG and total GSH in hDPCs after exposing to dental monomers without or with NAC for 24 h. Data represent the mean ± SD of three independent experiments (*n* = 3). *P < 0.05 vs. control group; #*P* < 0.05 vs. dental monomer-treated cells by one-way ANOVA. NS means no significance.

Compared to the control group, the intracellular ROS levels were increased in hDPCs after treatment with dental monomers for 6 h (all *P* < 0.05, Fig 2A). The over-production of ROS induced by dental monomers was significantly relieved by NAC treatment (all *P* < 0.05).

The GSH levels decreased after cells were treated with dental monomers (all *P* < 0.05, Fig 2B–2D). As expected, NAC alleviated monomer-induced depletion of GSH (*P* < 0.05 vs. dental monomer-treated cells). Compared to the control group, no difference in GSSG content was found after treatment with HEMA or MMA (*P* > 0.05, Fig 2B and 2C), and only a small decrease was found after treatment with TEGDMA (*P* < 0.05, Fig 2D).

**Fig 3:**
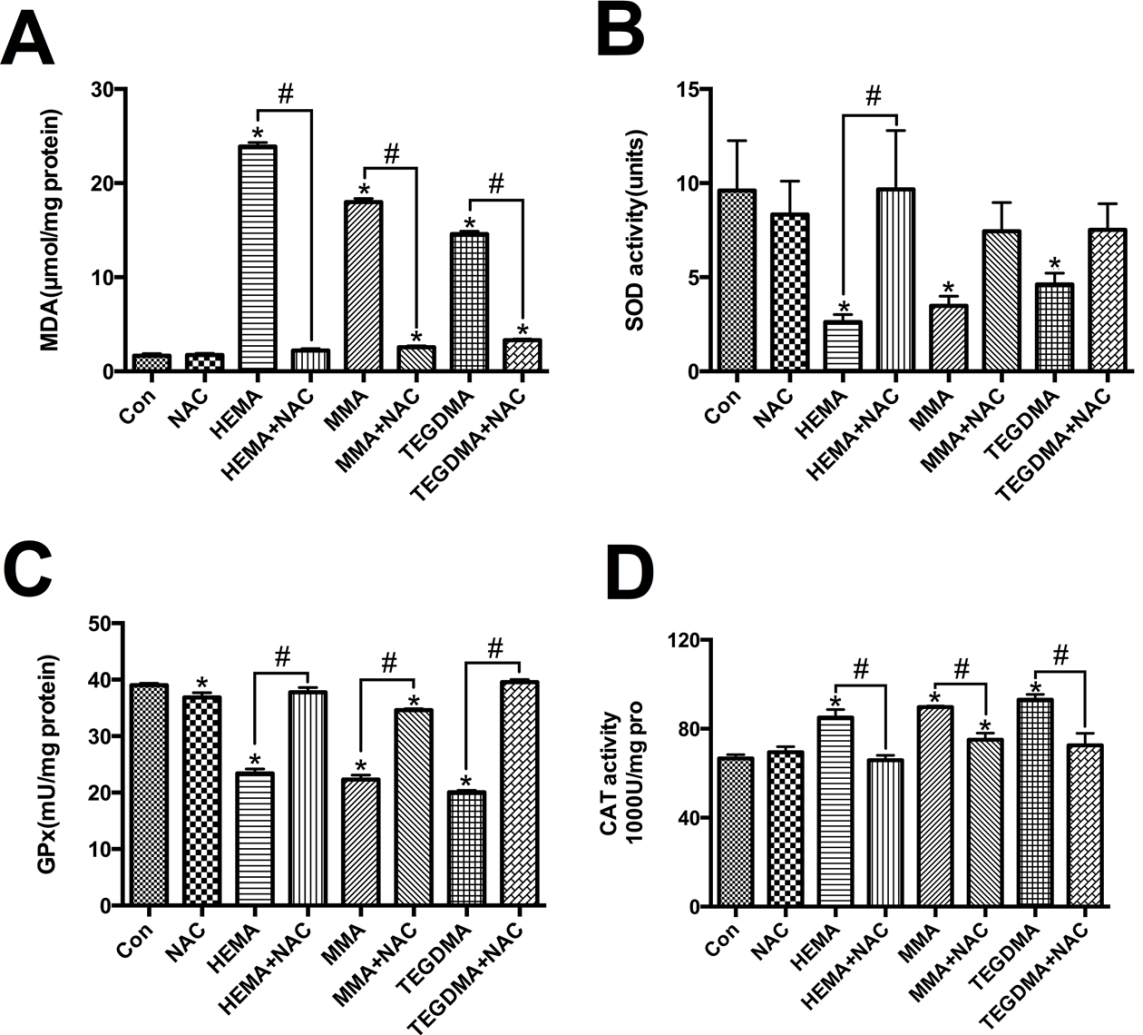
Dental monomers induced oxidative stress and differential changes of antioxidative enzymes. A–D. Levels of MDA (A) and activity of SOD (B), GPx (C), and CAT (D), in hDPCs exposed to dental monomers (1mM HEMA, 5mM MMA or 1mM TEGDMA) in the absence or presence of 10 mM NAC for 24 h. Data represent the mean ± SD of three independent experiments (*n* = 3). *P < 0.05 vs. control group; #*P* < 0.05 vs. dental monomer-treated cells by one-way ANOVA.

MDA level was remarkably increased after dental monomer treatment (all *P* < 0.05 vs. control group cells, Fig 3A). Compared to dental monomer-treated cells, NAC reduced intracellular MDA content level to almost the normal level (all *P* < 0.05).

SOD activity decreased after dental monomer treatment (*P* < 0.05 vs. control group cells, Fig 3B). Co-treatment of NAC with HEMA restored the decreased activity of SOD (*P* < 0.05 vs. HEMA-treated cells). For MMA and TEGDMA, NAC could also alleviate decreased activity of SOD, although there was no significant difference (*P* > 0.05 vs. dental monomer-treated cells). Similar results were observed for GPx activity (Fig 3C). CAT activity increased after 24 h of dental monomer treatment (all *P* < 0.05 vs. control group cells, Fig 3D). NAC co-treatment with monomers reduced CAT activity to an almost normal level (*P* < 0.05 vs. dental monomer-treated cells).

### Influences of dental monomers on apoptosis and the rescuing effects of NAC in hDPCs

**Fig 4:**
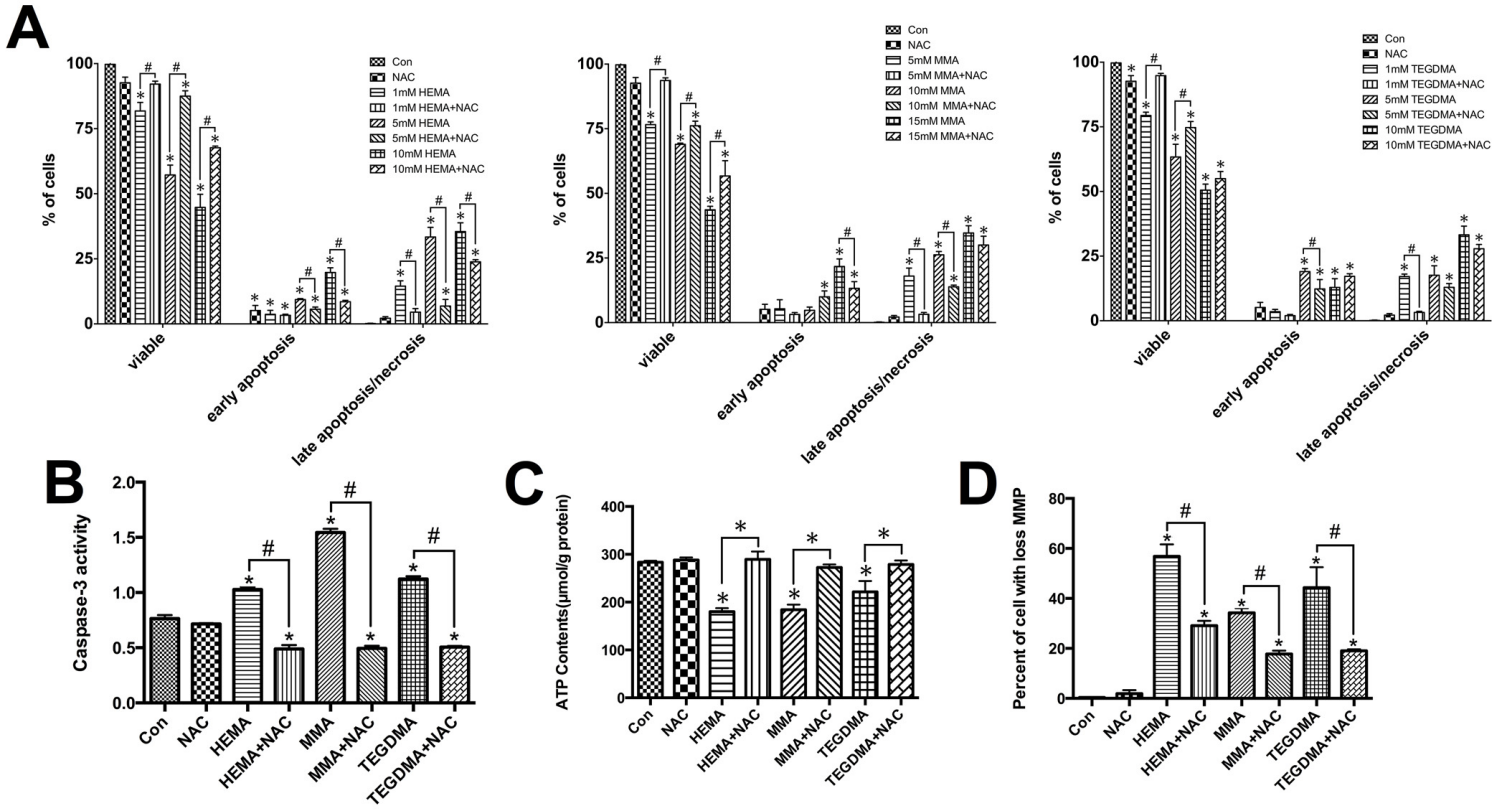
Dental monomers induced apoptosis. A. Apoptosis of hDPCs after exposure to HEMA (0-1-5-10 mM), MMA (0-5-10-15 mM) or TEGDMA (0-1-5-10 mM), in the absence or presence of 10 mM NAC for 24 h, as assayed by Annexin V and PI double staining. B–D. Influences of dental monomers (1mM HEMA, 5mM MMA or 1mM TEGDMA) and 10 mM NAC on caspase-3 activity levels (B), ATP level (C), and MMP (D). Data represent the mean ± SD of three independent experiments (*n* = 3). *P < 0.05 vs. control group; #*P* < 0.05 vs. dental monomer-treated cells by one-way ANOVA.

According to the results of Annexin V/PI staining, a dose-dependent decrease in the percentage of viable cells was observed in hDPCs exposed to dental monomers (Fig 4A). It was found that 10 mM NAC alone had nearly no influence on the viability of hDPCs (*P* > 0.05 as compared to the control group). Dental monomers greatly dose-dependently increased the percentage of cells in early apoptosis as well as in late apoptosis/necrosis. Quite the reverse, NAC exhibited restoring effects on cell viability and meanwhile decreased the percent of early apoptotic and late apoptotic/necrotic cells. Compared to untreated cells, there was no statistical difference in late apoptosis/necrosis in 1 mM HEMA, 5 mM MMA and 1 mM TEGDMA-treated cells in the presence of NAC (*P* > 0.05 as compared to the control group).

The occurrence of apoptosis was also confirmed by evaluating the activity of caspase-3, whose activation plays a central role in the execution-phase of cell apoptosis [16, 17]. As shown in Fig 4B, caspase-3 activity significantly increased after the treatment of dental monomers in hDPCs (*P* < 0.05 vs. control group cells). In contrast, co-treatment with NAC significantly inhibited dental monomer-induced activation of caspase-3 (*P* < 0.05 vs. dental monomer-treated cells).

### Influences of dental monomers and NAC on the functions and morphologies of mitochondria

Cellular ATP levels of hDPCs exposed to dental monomers, in the absence or presence of NAC, were demonstrated in Fig 4C. After 24 h of dental monomer treatment, ATP levels were statistically lower than those of the control groups (all *P*< 0.05, Fig 4C). Quite in contrast, NAC could restore the ATP levels in dental monomers-treated cells (all *P*> 0.05 as compared to the control group).

Next, we analyzed the effects of dental monomers and NAC on MMP, which was an important biomarker of intrinsic mitochondrial pathway. As can be seen in Fig 4D, the percent of hDPCs with collapsed MMP was significantly increased after treatment with dental monomers (all *P* < 0.05 vs. control group cells). NAC protected against dental monomer-induced depolarization of MMP (all *P* < 0.05 vs. dental monomer-treated cells).

**Fig 5:**
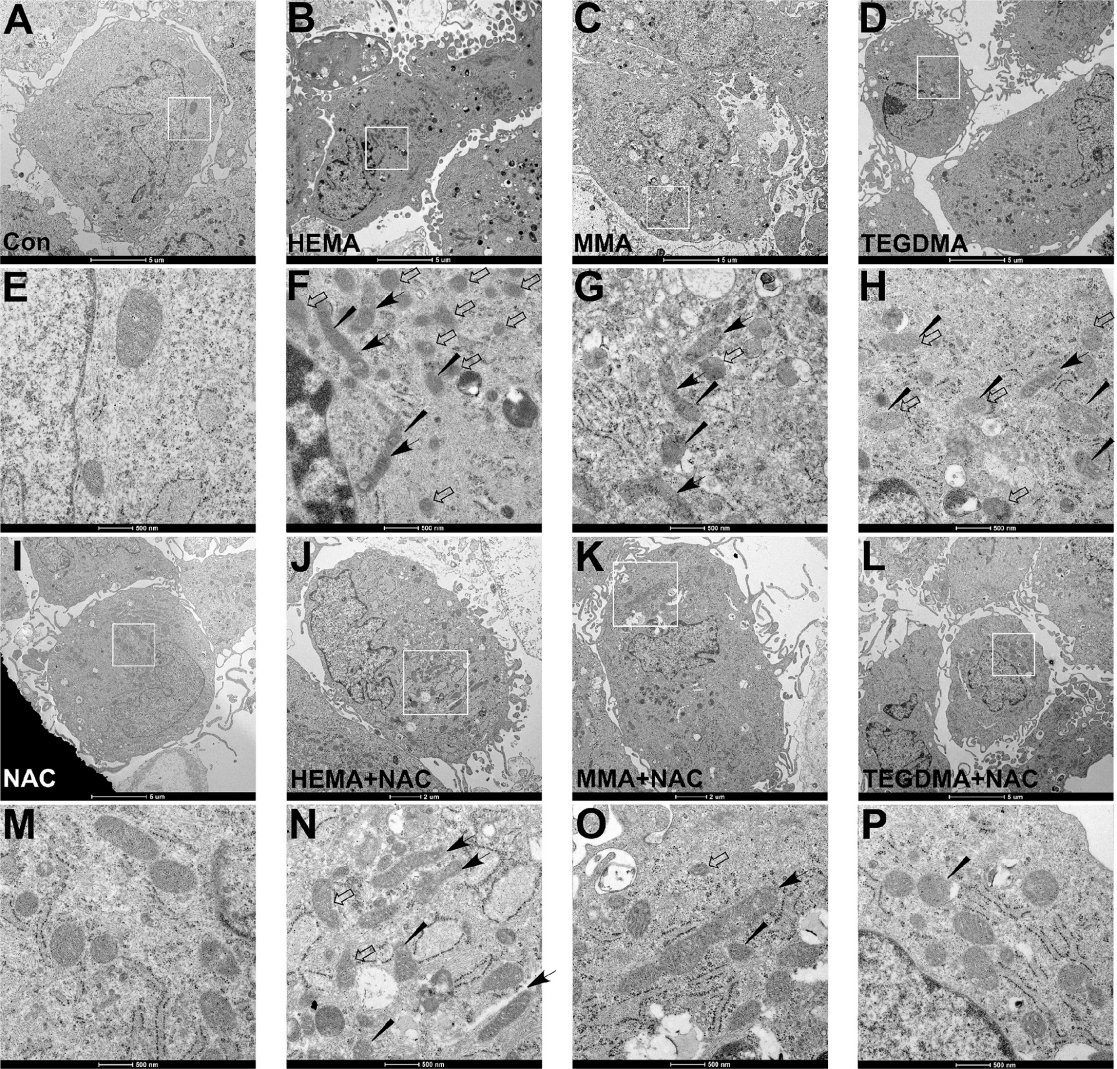
Changes of mitochondrial morphology observed by transmission electron microscopic (TEM). (A, E) Control hDPCs and (I, M) hDPCs treated with 10 mM NAC showing a well preserved morphology. (B-D, F-H) Following 24 h dental monomer treatment (1mM HEMA, 5mM MMA or 1mM TEGDMA), some cells showed nuclear and cytoplasmic signs of apoptosis (condensed/peripheralized nuclear chromatin and/or cytoplasm, cytoplasmic vacuolization). Larger and elongated mitochondria (black arrow) with impaired mitochondrial membrane integrity (white arrow), reduced number of cristae and deformed cristae (wedged) could be observed. (J-L, N-P) The presence of 10 mM NAC restored cell and mitochondrial morphology. Although larger and elongated mitochondria (black arrow), and deformed cristae (wedged) could still be observed, the structural integrity of mitochondrial membrane was preserved.

The morphologies of mitochondria in cells exposed to dental monomers, in the absence or presence of NAC, were examined by transmission electron microscope (TEM) here. After monomer treatment, some cells showed nuclear and cytoplasmic signs of apoptosis (condensed/peripheralized nuclear chromatin and/or cytoplasm, cytoplasmic vacuolization). As for the mitochondira, the control cells (Fig 5A–5E) and NAC-treated cells (Fig 5I–5M) showed integrity of mitochondrial membrane (inner and outer membranes), and the mitochondria cristae were not affected. However, the ultrastructures of dental monomers treated cells showed altered membrane structural integrity. Larger and elongated mitochondria with less number of cristae or deformed cristae were observed. Quite the reverse, the treatment with NAC could restore the membrane structural integrity of mitochondria in cells exposed to monomers, although some the mitochondria still showed deformed cristae (Fig 5J–5L, 5N–5P).

### Influences of dental monomers and NAC on key molecules of the intrinsic mitochondrial apoptosis pathway

**Fig 6:**
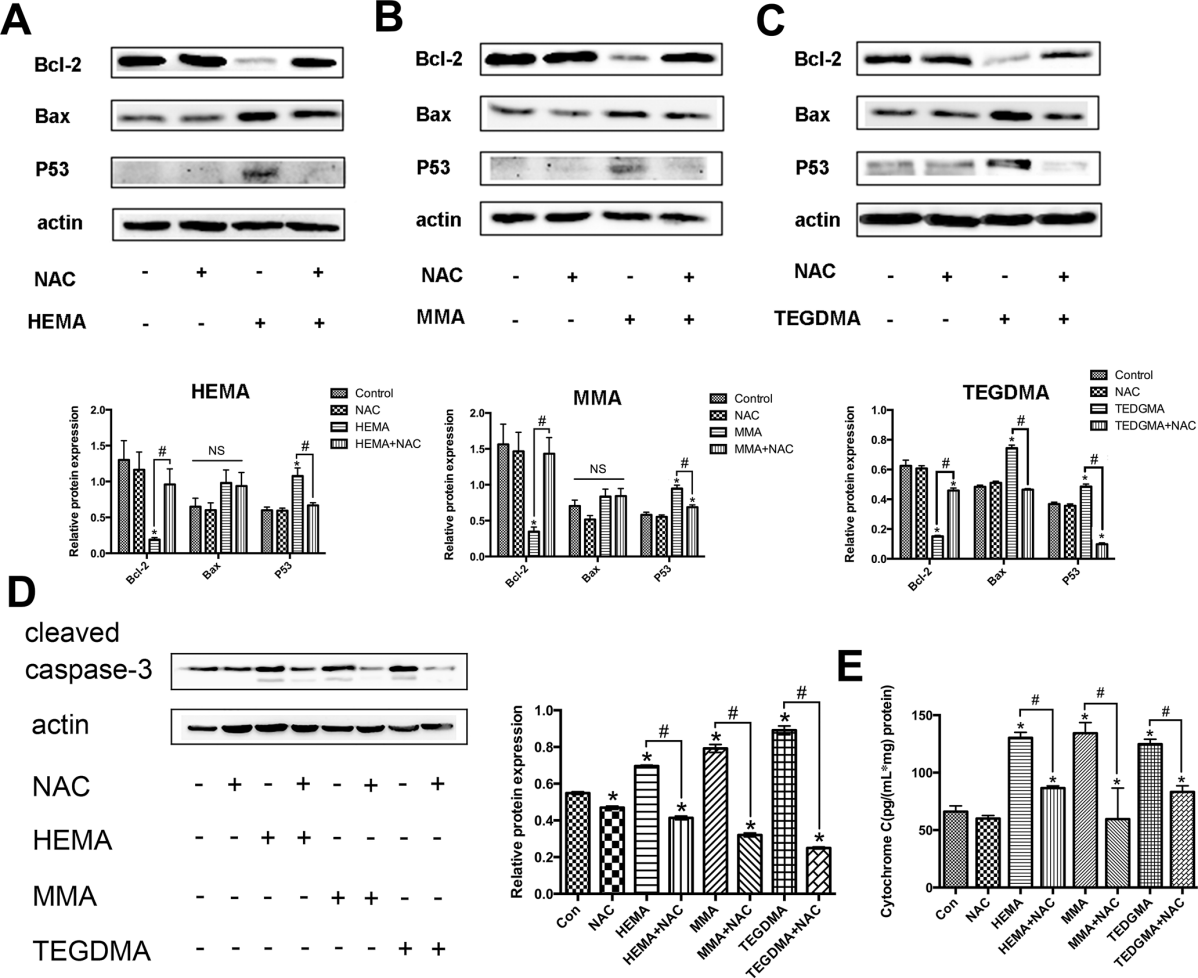
Dental monomers induced apoptosis through the intrinsic mitochondrial pathway. A–C. Expression levels of Bcl-2, Bax, and p53 in hDPCs after exposure to HEMA (1 mM) (B), MMA (5 mM) (C) or TEGDMA (1 mM) (D), with or without NAC (10 mM) for 24 h. D. Expression levels of cleaved caspase-3 in hDPCs exposed to HEMA (1 mM), MMA (5 mM) or TEGDMA (1 mM) in the absence or presence of NAC (10 mM) for 24 h. E. Levels of cytosolic Cyto C as assayed by ELISA. Data represent the mean ± SD of three independent experiments (*n* = 3). *P < 0.05 vs. control group; #*P* < 0.05 vs. dental monomer-treated cells by one-way ANOVA. NS means no significance.

Bcl-2 protein family is the key regulator of intrinsic mitochondrial apoptosis. Among the many members of Bcl-2 family proteins, Bcl-2 and Bax are two well-known molecules with anti-apoptotic, or proapoptotic effects, respectively [8, 18]. Western blot analysis revealed that in the presence of dental monomers, the expression of Bcl-2 was down-regulated (all *P* < 0.05 as compared to the control group, Fig 6A–6C), while the expression of Bax was up-regulated (*P* < 0.05 for TEGDMA as compared to the control group). The expression levels of Bax after HEMA or MMA treatment also showed a trend of increase, although there was no statistical significance compared to the control group (all *P* > 0.05). Co-treatment with NAC resulted in enhanced Bcl-2 expression (all *P* < 0.05 vs. dental monomer-treated cells), and reduced Bax expression (*P* < 0.05 for TEGDMA as compared to dental monomer-treated cells, and a marginal decrease without statistical significance for HEMA and MMA). p53 is a sensor of cellular stress and is a critical activator of the intrinsic mitochondrial apoptosis pathway. The p53-dependent regulation of apoptosis occurs through transcription dependent and transcription-independent mechanisms to either activate or in activate multidomain pro- and anti-apoptotic Bcl-2 proteins [7]. Dental monomer treatment significantly increased the expression of p53 (all *P* < 0.05 vs. control group cells) and NAC co-treatment significantly reversed dental monomer-induced up-regulation of p53. As for caspase-3, which is the executor of apoptosis, dental monomers greatly enhanced the intracellular level of cleaved caspase-3 (all *P* < 0.05 vs. control group cells, Fig 6D), whereas the presence of NAC could block the activation of caspase-3 (*P* < 0.05 vs. dental monomer-treated cells).

Cyto C is an important intermediate in intrinsic mitochondrial apoptosis. The release of Cyto C from mitochondria could activate caspase-9, which in turn activates caspase-3 and caspase-7 to execute cell death [19, 20]. Results of ELISA showed that the cytosolic Cyto C level was elevated when cells were exposed to dental monomers for 24 h (all *P* < 0.05 vs. control group cells, Fig 6E). The increase was blocked by co-treatment with NAC (*P* < 0.05 vs. dental monomer-treated cells). These observations indicate that NAC prevented the release of Cyto C from mitochondria into the cytoplasm after dental monomer treatment.

**Fig 7:**
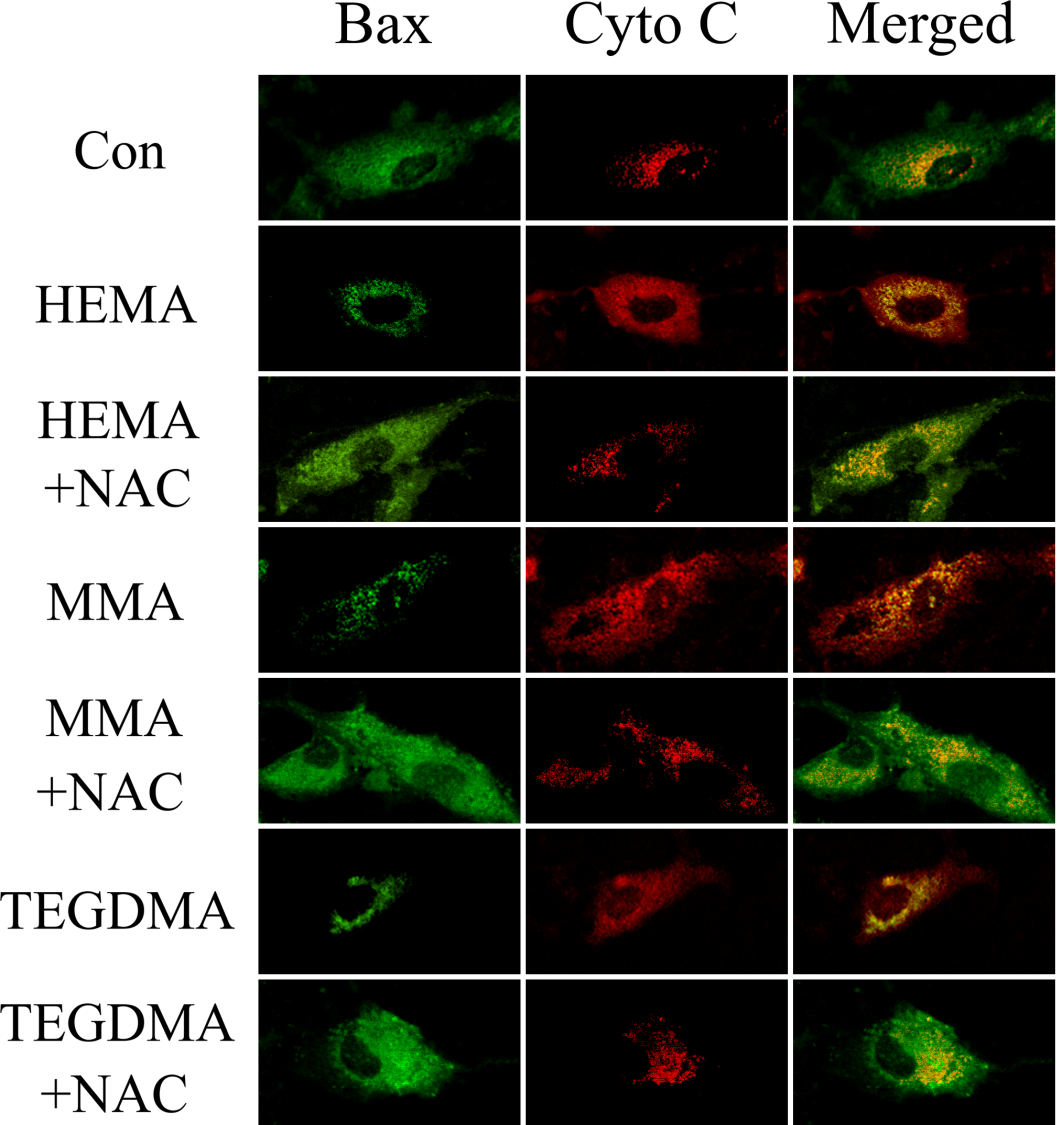
Double Immunofluorescence staining for Bax and Cyto C in hDPCs exposed to dental monomers in the absence or presence of NAC. G*reen fluorescence* represents Bax, whereas *red green fluorescence* represents Cyto C. *Yellow color* in the overlay of these two images indicates co-localization of Bax and Cyto C (presumably in mitochondria).

Immunofluorescence (IF) analysis revealed that Bax, which is diffusely present as fine granules in the cytosol of controls, translocated to mitochondria after dental monomer treatment with a punctate perinuclear distribution typical of mitochondria (Fig 7). In contrast, Cyto C, which was retained in the mitochondria in controls, redistributed diffusely in the cytoplasm (Fig 7). Quite the reverse, in the presence of NAC, Bax redistributed diffusely to cytosol, and meanwhile most of the strong spot signal of Cyto C was retained in the mitochondria in dental monomer-exposed cells (Fig 7).

## Discussion

In the present study, we demonstrated that dental monomers could disturb the intracellular redox balance, impair the functions of mitochondria and thus induced apoptosis through the intrinsic mitochondrial pathway in hDPCs. The presence of NAC could relieve dental monomer-induced oxidative stress and subsequently protect the cells from apoptosis.

### a. Dental monomer-induced oxidative stress in hDPCs

In our study, the results of CCK-8 assay revealed that dental monomers dose-dependently inhibited cell viability of hDPCs. However, the tested monomers revealed different level of toxic effects on hDPCs, with MMA showing the lowest toxicity, followed by HEMA and then TEGDMA. This result is in accordance with previous studies [21, 22]. The difference in the cytotoxicity of different dental monomers might be attributed to their chemical structures, such as the number of methacrylic groups, which can solubilize cell membrane and react with GSH to cause disturbance of intracellular redox balance [23–25].

The dental monomer-induced cytotoxicity was paralleled by over-production of ROS and depletion of GSH in hDPCs. This result is in accordance with previous studies, which indicate that oxidative stress is the primary mechanism for the toxic effects of dental monomers [26, 27]. GSSG is the product when reduced GSH react with oxidants [28]. If the reduction of GSH level is a secondary result caused by ROS overproduction, an increased level of GSSG should be expected. However, in our study, it was found that dental monomers reduced GSH level without significantly increasing GSSG level. Thus, it is believed that the reduction in intracellular level of reduced GSH after dental monomer treatment is not due to the oxidation of GSH to GSSG as a secondary result of ROS over-production. Rather, GSH may be directly depleted by the monomers. Indeed, several researchers reported that the methacrylic group of dental monomers could directly react with the thiol group of GSH through Michael-type addition reaction, and thus caused the depletion of GSH [29]. Under physiological conditions, there is a balance between ROS and antioxidative defense system [30]. However, when GSH, the key component of the antioxidative defense system, is depleted by dental monomers, ROS level could subsequently be elevated as a secondary result. When the formation of ROS during monomer exposure is beyond the capacities of anti-oxidative mechanisms, the overproduced ROS can react with cellular macromolecules, such as lipids, proteins and DNA, and detrimental damages can be resulted. MDA is the results from lipid peroxidation of polyunsaturated fatty acids, and is deemed as an indicator of oxidative damage [31]. Our data showed that MDA generation drastically increased after treatment with dental monomers, indicating that oxidative damage was generated after hDPCs were exposed to dental monomers for 24 h.

Besides the non-enzymatic antioxidant GSH, enzymatic antioxidants directly control cellular redox homeostasis by regulating the levels of particular ROS. In this study, CAT activity was increased after exposure to dental monomers, whereas SOD and GPx activities were reduced. Functionally, SOD catalyzes the breakdown of O_2_^-^ into O_2_ and H_2_O_2_, whereas CAT and GPx subsequently conduct the conversion of H_2_O_2_ to H_2_O and O_2_ [32, 33]. A recent study revealed that H_2_O_2_ was the major sort of ROS after dental monomer treatment [34]. Because GPx requires GSH as a substrate to reduce H_2_O_2_, the limited source of the substrate GSH as a consequence of monomer-induced GSH depletion can subsequently restrict the activity of GPx, causing the observed reduction in GPx activity [33]. The elevated H_2_O_2_ formation may lead the increased CAT activity and decreased SOD activity levels as a result of a feedback regulation [35].

### b. Dental monomers impaired the functions of mitochondria and induced intrinsic mitochondrial apoptosis in hDPCs

In this study, a dose-dependent decrease in the percentage of viable cells, as well as an increase in apoptotic and necrotic cells was observed in hDPCs exposed to dental monomers. Furthermore, dental monomers enhanced the activity of caspase-3. These data is in accordance with many previously published researches [3, 34]. Although it is well accepted that dental monomers can cause apoptosis, its detailed pathway is still largely unknown. Apoptosis is a complex process characterized by cell shrinkage, chromatin condensation and internucleosomal DNA fragmentation [36]. Considering that the over-produced ROS after dental monomer treatment can attack DNA and cause DNA double strand breaks (DSBs), which may subsequently activate the stress sensor p53, we hypothesized that dental monomer-induced apoptosis may occur through a p53-related pathway. Thus we investigated the changes of p53 level after dental monomer treatment and found that p53 expression was remarkably elevated. Besides its anti-tumor activity, p53 is also a key element in the intrinsic mitochondrial apoptosis pathway [7, 8]. It regulates intrinsic mitochondrial apoptosis through transcription-dependent and transcription-independent mechanisms to either activate or inactivate multidomain pro- and anti-apoptotic Bcl-2 proteins. Therefore, we subsequently hypothesized that p53-related intrinsic mitochondrial apoptosis pathway might be involved in dental monomer-induced cell death. Therefore, the morphologies and functions of mitochondria were studied in details. TEM observation indicated that dental monomers had very destructive effects on mitochondria. Several larger and elongated mitochondria with unclear mitochondrial membrane, less cristae or deformed cristae were observed. The changes in mitochondrial morphology were accompanied with a decreased level of ATP. Furthermore, disruption in the integrity of mitochondrial membrane, inevitably leaded to depolarization of MMP and subsequent Cyto C release from mitochondrial membrane into the cytoplasm.

Based on these results, we then moved on to study the expression level of other key molecules of the intrinsic mitochondrial apoptosis pathway to support our hypothesis. The results of Western Blot analysis revealed that dental monomers induced upregulation of the pro-apoptotic protein Bax and cleaved caspase-3, as well as the downregulation of the anti-apoptotic protein Bcl-2. The intrinsic mitochondrial apoptosis pathway had been well studied by numerous studies [34, 37]. Apoptosis factors acting on the mitochondria are triggered with mitochondrial membrane damage, reduction in MMP, and deficiency of respiratory chain. The apoptotic factors of Bcl-2 family play an important role in regulating the expression of anti-apoptotic and pro-apoptotic members of the Bcl-2. After cells receive apoptosis signal, Bcl-2 family proteins change their usual localization and target patterns from the cytoplasm to the mitochondrial membrane, trigger mitochondrial dysfunction, Cyto C release and caspase activation, and eventually lead to cell death [38]. Indeed, our immunofluorescence analysis revealed that after dental monomer treatment, Cyto C was released from the mitochondria to cytoplasm, while Bax translocated from cytoplasm to mitochondria. The observed activation of caspase-3, impaired mitochondria function, changes in p53, Bcl-2, Bax and translocation of Bax and Cyto C, supported our hypothesis that the intrinsic mitochondrial pathway might be involved in dental monomer-related cytotoxicity.

### c. NAC reduced dental monomer-induced oxidative stress and blocked the apoptotic effects in hDPCs

NAC is a well-known free radical scavenger that can readily enter the cells. We found that NAC could alleviate dental monomer-induced oxidative stress and thus protected the cells from apoptosis. The protective effects of NAC may be attributed to three aspects. First, it can directly scavenge the over-produced ROS. Second, as a cysteine-donating compound, NAC acts as a cellular precursor of GSH to replenish the depleted GSH pool after dental monomer treatment [39]. Third, according to recent publications, NAC may also directly react with the methacrylic group of dental monomers through Michael-type addition reaction, and thus reduce the availability of free dental monomers [40–42]. In a recent review, which discussed the mechanisms underlying adaptive cell responses to dental monomer-induced oxidative stress, the authors suggested that the formation of adducts with dental monomers could only be partially responsible for the protective effects of NAC against monomer-induced cytotoxic effects [35]. Some researchers, using GSH synthesis-modulating substances such as GSH synthesis inhibitor buthionine sulfoximine (BSO) and, GSH synthesis promotor 2-oxo-4-thiazolidine-carboxylic acid (OTC), found that the intracellular GSH is the primary antioxidant central to the regulation of cell response towards oxidative stress induced by dental monomers [14, 34]. In addition, OTC showed similar protective effects on dental monomers-induced cytotoxicity as NAC. These results indicate that, the protective effect of NAC against monomer-related toxicity might be mainly attributed to replenish the depleted GSH pool. However, the detailed mechanism underlying NAC protection against dental monomer-induced cytotoxicity needs further investigation.

## Conclusions

**Fig 8:**
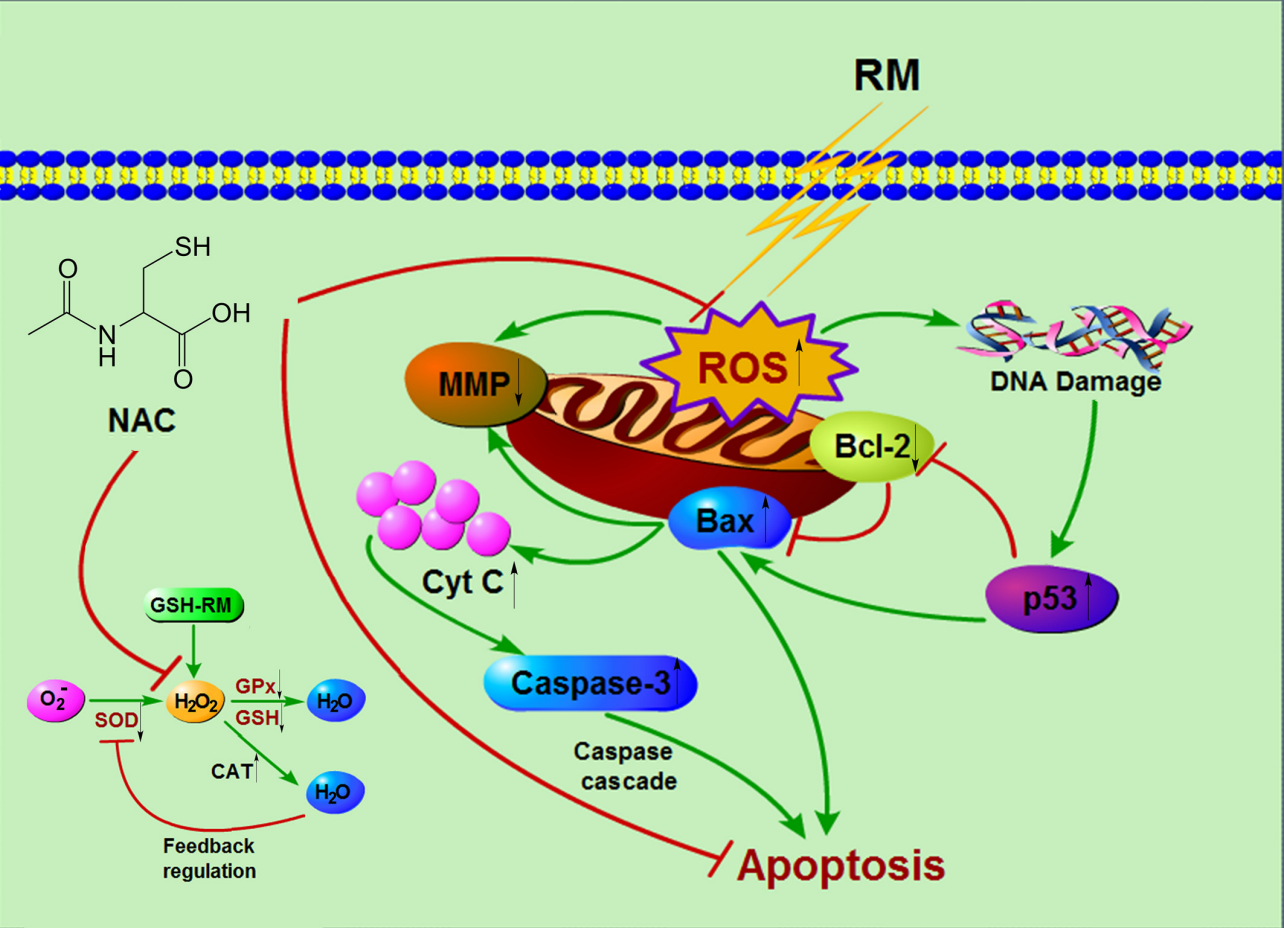
A schematic diagram summarizing the redox regulation, the induction of intrinsic mitochondrial apoptosis and rescuing effects of NAC in cells exposed to dental resin monomers (RM). Dental resin monomers can chemically react with the key non-enzymatic antioxidant GSH and lead to the depletion of intracellular GSH pool. This can subsequently lead to the over-production of ROS, especially H_2_O_2_. The depletion of GSH and over-production of H_2_O_2_ can induce a decrease in GPx activity and increase in CAT and SOD activity as a result of a feedback regulation. Oxidative stress beyond the capacities of cellular redox regulation causes oxidative DNA damage, which subsequently upregulates the stress sensor p53 and thus triggers intrinsic mitochondrial apoptosis. NAC can alleviate dental monomer-induced oxidative stress and thus protect the cells from apoptosis by scavenging the over-produced ROS, replenishing GSH pool and reacting with the dental resin monomers.

The conclusions from the present findings are summarized in a hypothetical model (Fig 8). With the limitations of the present *in vitro* study, it might be concluded that dental monomers caused disturbance of intracellular redox balance, which is characterized by depletion of GSH, over-production of ROS, and differential changes of antioxidative enzymes. The oxidative stress subsequently impaired mitochondria function, up-regulated p53, and eventually initiated the intrinsic mitochondrial apoptosis pathway. NAC remarkably relieved dental monomer-induced oxidative stress and subsequently protected the cells against apoptosis.

## Supporting Information

S1 Raw Data (XLS)
